# Financial Report of the Hahnemann Monumental Committee

**Published:** 1899-11

**Authors:** 


### FINANCIAL REPORT OF THE HAHNEMANN MONUMENTAL COMMITTEE.

Nov. 8th, 1899.

RECEIPTS.

| | | |
|---|---|---|
| From subscriptions, interest on deposit, sale of models, etc...... | | $29,233.84 |

EXPENDITURES.

| | | |
|---|---|---|
| Contract for building, account of.. | $25,000.00 | |
| Contractor, expenses of .......... | 191.10 | |
| Awards, competitive designs ...... | 721.73 | |
| Models ......................... | 525.15 | |
| Photographs .................. | 117.91 | |
| Printing, circulars, booklets, stationery ................... | 829.94 | |
| Postage ....................... | 433.50 | |
| Clerical assistance ............... | 880.90 | |
| Expressage and freight ........... | 29.50 | |
| Railroad fares.................... | 220.11 | |
| Incidentals, telegrams, collections, commissions ............... | 52.63 | |
| Auxiliary committees, expenses of.. | 119.06 | |
| Cash on hand.............. | 112.31 | $29,233.84 |

New York, Nov. 28th, 1899.

Editor of The Homœopathic Physician.—The Finance Committee appointed by the American Institute at their meeting at Atlantic City, in June, of this year, have received from the Monument Committee the foregoing report of the present financial condition of the project. This report extends over a period of nearly eight years and will show the receipts and expenditures during that period. The committee requests its publication in order that your readers may have an opportunity to examine it and appreciate the large amount of work that has been done by the Monument Committee. It is estimated that $25,000 more will be required to place the monument upon its pedestal, and plans are now maturing that it is hoped will result in rapidly providing that amount.

Will you kindly give this space in your next number.

Very truly yours,
The Finance Committee.

Geo. G. Shelton, chairman; 521 Madison avenue, New York; O. S. Runnels, M. D., secretary, 203 N. Meridian street, Indianapolis, Ind.; Benjamin F. Bailey, M. D., Lincoln, Nebraska; John R. Kippax, M. D., 3154 Indiana avenue, Chicago, Ills.

